# Brazilian XP-E siblings carrying a novel *DDB2* variant developed early-onset melanoma: a case report

**DOI:** 10.1186/s12920-023-01622-8

**Published:** 2023-08-12

**Authors:** Ana Rafaela de Souza Timoteo, Isabel Cristina Pinheiro de Almeida, Andrey A Yurchenko, Sheila Ramos de Miranda Henriques, Paulo de Souza Segundo, Fatemeh Rajabi, Sergey Nikolaev, Tirzah Braz Petta

**Affiliations:** 1 Departamento de Biologia Celular e Genética, Universidade Federal do Rio Grande do Norte, Av. Senador Salgado Filho, s/n, Natal, 59078-970 RN Brazil; 2 Hospital Liga contra o Câncer, Av. Miguel Castro, Natal, 1355, 59062-000 RN Brazil; 3 Cancer Genomics Lab, B2M, Gustave Roussy Cancer Campus, 114 rue Edouard Vaillant, Villejuif, 94805 France; 4 Hospital Universitário Onofre Lopes, Universidade Federal do Rio Grande do Norte, Av. Nilo Peçanha, 620, 59012-300 RN Natal, Brazil; 5 Keck School of Medicine, Department of Pathology, University of Southern California, HMR 315, 2011, Zonal Avenue, Los Angeles, CA 90089-9092 USA

**Keywords:** Case Report, Xeroderma Pigmentosum, XP-E, Melanoma

## Abstract

Xeroderma pigmentosum group E (XP-E) is one of the least common forms of XP, a rare syndrome where patients are prone to develop skin cancer in exposed sunlight areas. XP-E patients are generally not diagnosed until they are adults due to the mild phenotype. Case presentation: two XP-E siblings, female, 23 years, and male, 25 years, from a Brazilian consanguineous family carrying the novel missense pathogenic variant in *DDB2* gene, NM_000107.3:c.1027G > C, associated with skin cancer early-onset and severe phenotype, as nodular melanoma in the cornea and in the ear. Conclusion: The assessment of genomic variant pathogenicity was a challenge since this family belongs to an underrepresented population in genomic databases. Given the scarcity of literature documenting XP-E cases and the challenges encountered in achieving an early diagnosis, this report emphasizes the imperative of sun protection measures in XP-E patients. Additionally, it highlights the detrimental impact of the COVID-19 pandemic on cancer diagnosis, leading to the manifestation of a severe phenotype in affected individuals.

## Background

Xeroderma pigmentosum (XP) is characterized by increased sensitivity to ultraviolet radiation-induced sunburn, skin cancers, ocular disease, and neurological degeneration [1]. Patients with XP have a 2,000-fold increased risk of melanoma and a 10,000-fold increased risk of nonmelanoma skin cancer XP [2]. XP is classified into eight genetic groups, according to mutated gene: groups XP-A to XP-G presenting alteration in *XPA, ERCC3, XPC, ERCC2, DDB2, ERCC4, and ERCC5* genes, respectively, and the group XP-V is characterized by mutations in *POLH* [3]. The diagnosis is primarily clinic-based and confirmed by genetic tests or by functional cell-based methodologies.

XP-E is a rare form of the disorder, with a few reported cases in which patients with pathogenic variants at the *DDB2* gene result in absent or dysfunctional *DDB2* (damage-specific DNA-binding protein 2, also known as p48 subunit) [4, 5]. The human *DDB2* gene, located on chromosome 11 (11p11.2), consists of 10 exons encoding a 48 kDa protein of 427 amino acids exclusively localized in the nucleus and forms a heterodimer with DDB1 forming UV-DDB complex. DDB2 plays a central role in Global Genomic Nucleotide Excision Repair (GG-NER) in recognizing DNA damage [4, 6]. DDB2 binds to pyrimidine dimers including isomers of CPD (cyclobutane pyrimidine dimers) and 6-4PP (6 − 4 pyrimidine-pyrimidone photoproducts) with a high affinity and specificity.

In this article, we share the compelling clinical journey of two siblings, and we have obtained the necessary consent from both the patients and their parents to showcase their images. The rationale behind this decision stems from the fact that the lesions affect their face and eye areas, making the inclusion of pictures immensely valuable in accurately describing the tumors.

**Fig. 1:**
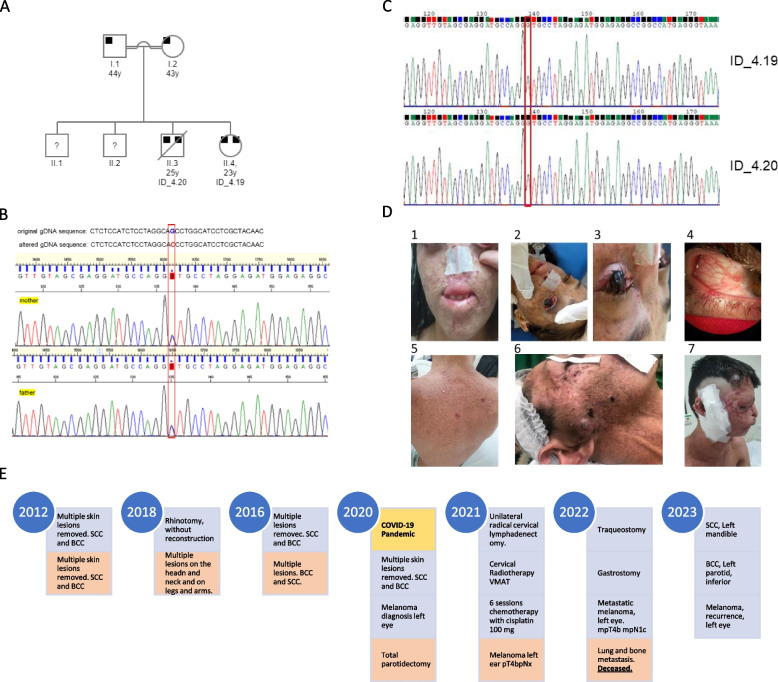
** A** Pedigree of the family showing consanguineous marriage. Both parents (I.1 and I.2) are heterozygous for NM_000107.3:c.1027G > C and patient ID_4.19 (II.4) and patient ID_4.20 (II.3) are homozygous for this variant. The two older siblings are asymptomatic and were not tested. **B** Reverse sequence electropherogram from mother and father of siblings. The NM_000107.3:c.1027G > C variant is highlighted in red. **C** Reverse sequence electropherogram from ID_4.19 and ID_4.20. **D** Photos to evidence severe phenotype. Patient ID_4.19 1. Photo from exeresis to remove lesions in lips, face and nose. 2 and 3. Photo of the nodular, pigmented and elevated lesion of the LE, extending throughout the cornea. 4. Photo showing the melanoma of the RE. Patient ID_4.20 5. Photo showing lesions that were removed and pigmentation on the back, exposed to sunlight. 6. Photo from exeresis of the lesion located in the right parotid gland, before surgery and 7. After the total parotidectomy. **E** Milestones of patient’s clinical history, Patient ID_4.19 is highlighted in blue and for Patient ID_4.20, in orange, unfortunately, he passed away on 12/06/2022

The two siblings with a clinical diagnosis of XP, a 20-year-old female (ID_4.19) and a 22 years-old male (ID_4.20) fourth and third child of consanguineous parents (Fig. 1A). They live in a highly consanguineous region with a high prevalence of rare genetic diseases, named Seridó, in the Rio Grande do Norte state, Brazil [7]. They both had the first diagnosis of skin carcinoma at an early age when she was 13 years old and he was 15 years old, not common for XP-E. Both were submitted to multiple surgeries to remove skin lesions in sun-exposed areas. The patient ID_4.19 had removed 19 BCC and 20 SCC and patient ID_4.20 had removed 10 BCC and 25 SCC. Both have early diagnosis of metastatic melanoma at the age of 23 years.

## Case presentation

1. The Patient ID_4.19 is female, 23 years (Born in 1999). No neurological abnormalities detected. Her skin presents punctate, flat, pigmented lesions on the eyelids, face, and all UV-exposed areas of the body. She is being followed at the Head and Neck Department since 2012 and had multiple skin lesions removed (Table 1). In May 2018, she underwent a rhinotomy, without reconstruction (Fig. 1C1). Both eyes showed an extensive pigmented lesion on the entire corneal surface, presenting visual acuity of the right eye (RE) 20/100 and left eye (LE) with vision impairment. She underwent an excisional biopsy for a corneal lesion in the LE, diagnosed as SCC and this pigmented lesion was elevated, extending throughout the cornea, making it impossible to assess the internal structures of the eye (Fig. 1C2 and 1C3). She reported a progressive increase of the lesion for the last 5 years when she was in use of topical Mitomycin therapy, however, she lost follow-up and only came back in 2020, after the evolution of the lesion and vision loss of the LE (Fig. 1C4). She underwent evisceration surgery of the LE and the result of corneal biopsy was a nodular melanoma, Breslow thickness 5,5 mm, mitotic index 4/1,0mm2 and ulceration present. The pathology report showed a free/negative tumor resection margin. Her RE had a SCC lesion and she was treated with topical Mitomycin, with a follow-up with the ophthalmologist. During the COVID-19 pandemic she lost follow-up again and she came back in August 2021 with an expansive and dark lesion on the left eyeball with extraorbital extension, measuring 4,9 × 2,8 × 3,4 cm. The diagnosis was recurrent or residual melanoma, she underwent an additional evisceration surgery. The nodular melanoma showed Breslow thickness 30,0 mm, Clark 5, TIL (tumor infiltrating lymphocytes) and ulceration present, mitotic index 11/1,0 mm2. She was treated with radiotherapy and 6 cycles of cisplatin 100 mg, currently under observation for new lesions and followed by the oncologist.
2. The Patient ID_4.20 is male, 25 years (Born in 1997). No neurological abnormalities detected. He presents multiple lesions in areas exposed to UV, such as his back (Fig. 1C5), legs, arms, and face (ears, nose and lips). He is being followed at the Head and Neck Department since 2012 and had multiple skin lesions removed (Table 1). In February 2020, he was submitted to total parotidectomy on the right side to remove a neuroendocrine carcinoma, carcinoid type (Fig. 1C6 and 1C7). He did not present with vision impairment. He lost follow-up during the COVID-19 pandemic and came back to follow-up in February 2021, at 25 y, with a suspicious lesion in the left ear, measuring 1,7 × 0,8 cm. The diagnosis was nodular melanoma, with 8,0 mm, mitotic index 5/1,00 m2, TIL and ulceration present. The immunohistochemistry result showed stage is pT4bpNx, Breslow thickness 8,0 mm, Clark 5, IHC profile, S100+, MART-1+, p63-, cytokeratin- and EpCAM-. At the same time, he also had two extensive lesions at the parathyroid (3,2 × 2,4 cm and 2,2 × 1,8 cm), that was extended to the cervical region with nerve compression and neuropathic pain in the right side. Stellate ganglion block was made using analgesic treatment with buprenorphine (5 mg) and pregabalin (75 mg). In September 2022, the CT image analysis showed lung and bone metastasis. He had treatment with cisplatin for the malignant melanoma and due to toxicity, his performance status was low he was admitted to the ICU for a gastrostomy e tracheotomy, and now is under palliative care. Unfortunately, he passed away on 12/06/2022.

**Table 1:** Number of lesions according to carcinoma type Basal Cell Carcinoma (BCC) and Squamous cell carcinoma (SCC).

Patient ID	Current age (Years-old)	Age of 1st symptoms	BCC (N)	SCC (N)	Melanoma age at onset
ID_4.19	23	13	18	19	20
ID_4.20	25	15	13	22	22

### Diagnostic assessment: genetic analysis

The surgical lesion tissue of each sibling was stored in Allprotect® Tissue Reagent (Qiagen) and DNA was extracted with DNeasy Blood & Tissue Kit (Qiagen). WES was performed using the Agilent SureSelect v6 and BGISEQ500 sequence platform to the coverage 50X, the mutations were identified using GATK4 according to the GATK Best Practices Protocols [8]. The BCFtools/RoH command [9] was used to detect runs of homozygosity (RoH) in sequencing data using Markov model (parameters: minimum length 4 Mb; minimum number of consecutive markers = 10). DNA was extracted from saliva from the parents. For Sanger sequencing, we used 100 ng of DNA and XP-E primers with the Platinum™ PCR SuperMix High Fidelity from Thermo Fisher, according to the manufacturer. The left primer starts at position 49 and the right primer at position 232, considering the version of the genome GRCh37.p13. The primers sequences are Forward, GGGAGGCCAGCTAGGGATTA and Reverse, CTCACCGAACTGATGCCAGA.

**Fig. 2:**
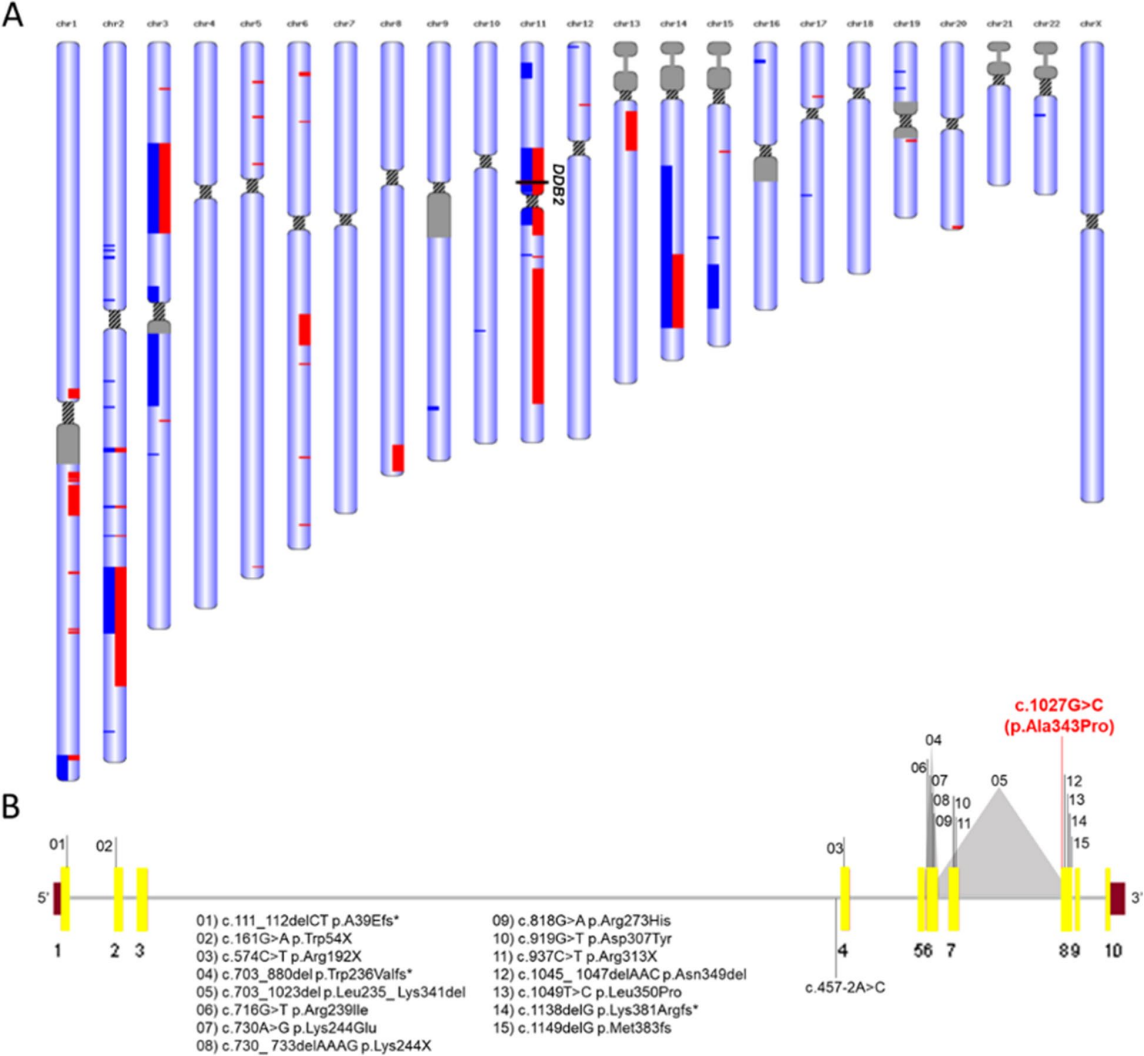
Genetic analysis of the WES. **A** XP-E patients ROH regions detected at each chromosome. The regions highlighted in blue and in red refer to ROH in Patient ID_4.20 and Patient ID_4.19, respectively. **B** *DDB2* gene schematic representation and pathogenic variants and VUS described in databases. The exons are in yellow, all variants submitted at ClinVar and described in literature are listed (reviewed by Yang and coworkers, 2020 [26] and the variant detected in the two siblings is highlighted in red

A region with 123 Mb in runs of homozygosity (RoH) was identified, which corresponds to 4% of the genome and confirms consanguinity (Fig. 2A). A 25 Mb region was on chr11 36 Mb − 61 Mb where *DDB2* gene (NM_000107) is located (chr11:47,236,525 − 47,260,768). The quality score of the variant is high: 4656.03 and the coverage for ID_4.19 was 127x and for ID_4.20, 38x. In both patients, we found the variant in *DDB2* c.1027G > C, at position chr11:47259391 in exon 8 resulting in p.A343P amino acid change, therefore NM_000107.3:c.1027G > C. *DDB2* homozygous missense variants are known to cause Xeroderma Pigmentosum group E (MIM: 278,740). This variant was not found in the general population (1000Genomes [10], dbSNP151 [11], GNOMAD [12]) or in disease-associated database CLINVAR [13], LOVD [14]. Both patients carry a homozygous variant and the same variant was detected in heterozygosity in their parents by Sanger sequencing (Fig. 1A and B). The function prediction analysis to identify disease-relevant nonsynonymous Single-Nucleotide Variants was performed using the MCAP [15] and REVEL [16] tools which rendered the scores 0.07 and 0.26 respectively, suggesting that the variant may be pathogenic, but the pathogenicity score is not high. According to a decision tree model that measures and compares the accuracy with four known mutation predictors and seventeen supervised machine-learning algorithms, the variant NM_000107.3:c.1027G > C is pathogenic [17]. Additionally, this G in position c.1027, located in exon 8, is highly conserved among humans and other hominids (Altai Neandertal G/G; Denisova G/G; Vindijia Neandertal G/G). According to the ACMG (American College of Medical Genetics) and to the AMP (Association for Molecular Pathology) guidelines [18], variant segregation with diseases in multiple family members supports evidence of pathogenicity when a gene is known to be associated with the disorder. WES data is available from the corresponding author after reasonable request due to the privacy of patient information data.

## Discussion and conclusions

In this paper, we present two XP-E patients from a Brazilian consanguineous family affected with early-onset multiple skin lesions and malignant melanoma. XP-E is conventionally described as a mild XP phenotype, with residual levels of photoproducts DNA repair (between 50 and 100%), which makes the diagnosis difficult and generally, they are not diagnosed until adulthood [19, 20]. The reason for the phenotypic variations in XP-E patients may depend on environmental exposure and lifestyle. During the undiagnosed period, they accumulate large quantities of pre-carcinogenic lesions, and as a consequence, they may develop hundreds of skin cancers [21]. Unfortunately, the COVID-19 pandemic has difficult access to healthcare [22], especially in vulnerable rural areas, resulting in the evolution of the lesion into melanoma.

The parents are heterozygous and asymptomatic and 2 siblings from 4 are homozygous for the variant c.1027G > C (p.A343P), in *DDB2* gene. This variant was not found in the general population from public databases. Despite the novel variant pathogenicity score is not extremely high, the position of modification seems to be extremely important to DDB2 DNA-binding function. DDB2 is ubiquitously present in human tissues and localizes ahead of XP-C to repair CPD and 6-4PP lesions in DNA [6]. One significant challenge we faced is the limited data of underrepresented populations in genomic databases and this continuous lack of diversity poses unique difficulties when it comes to accurately determining the pathogenicity of genomic variants. Genomic variant pathogenicity is typically evaluated by comparing the observed variants to known variants cataloged in databases. These databases primarily consist of genomic data from individuals of European ancestry, with a disproportionately smaller representation of other ethnic groups. As a result, the majority of our current understanding of variant pathogenicity is biased towards European populations. Without an adequate representation of diverse populations, we may miss crucial insights into variant pathogenicity and fail to provide accurate diagnoses, risk assessments, and tailored treatment plans for individuals from underrepresented backgrounds.

In a study with 89 XP patients, it was observed a specific propensity to ocular problems in XP-C group compared with XP-E and XP-V patients, while these two last groups had more skin cancers [23]. The patient ID_4.19 presented lesions in both eyes, with nodular melanoma in LE cornea, and residual/recurrent disease after one year. The etiology of ocular problems in XP-E patients is not well known since there are few reports.

This report presents the consequence of high UV light incidence in the XP-E outcome in patients living in a rural area in a tropical country. In the region Serido, UVA/UVB incidence is extremely high, therefore increasing the frequency of pyrimidine dimers [24, 25], in addition to this environmental risk, these patients lost follow-up during the COVID-19 pandemic, these two factors can explain, respectively, the anticipation and severity of the clinical manifestation observed in these 2 patients, with melanoma diagnosis at 20 and 22 years.

## Data Availability

The datasets used and/or analyzed during the current study are available from the corresponding author on reasonable request. Datasets will be publicly freely available at the time the primary manuscript is accepted for publication.

## Abbreviations

1000Genomes: The 1000 Genomes Project
ACMG: American College of Medical Genetics and Genomics
AMP: Association for Molecular Pathology
BCC: Basal Cell Carcinoma
CPD: Cyclobutane Pyrimidine Dimers
dbSNP151: The Single Nucleotide Polymorphism database
DDB2: Damage-specific DNA-binding protein 2
GG-NER: Global Genomic Nucleotide Excision Repair
GNOMAD: Genome Aggregation Database
Mb: Megabase
CT: Computer Tomography
ICU: Intensive Care Unit
LOVD: Leiden Open Variation Database
RoH: Runs of homozygosity
SCC: Squamous Cell Carcinoma
TIL: Tumor-infiltrating Lymphocytes
UVA: Ultraviolet A
UVB: Ultraviolet B
XP: Xeroderma pigmentosum
WES: Whole Exome Sequencing
6-4PP: 6 − 4 pyrimidine-pyrimidone photoproducts
